# Association Between Expanded Genomic Sequencing Combined With Hearing Screening and Detection of Hearing Loss Among Newborns in a Neonatal Intensive Care Unit

**DOI:** 10.1001/jamanetworkopen.2022.20986

**Published:** 2022-07-11

**Authors:** Yunqian Zhu, Liyuan Hu, Lin Yang, Laishuan Wang, Yulan Lu, Xinran Dong, Tiantian Xiao, Zhengmin Xu, Bingbing Wu, Wenhao Zhou

**Affiliations:** 1 Department of Neonatology, Children’s Hospital of Fudan University, National Children’s Medical Center, Shanghai, China; 2 Department of Pediatric Endocrinology and Inherited Metabolic Diseases, Children’s Hospital of Fudan University, National Children’s Medical Center, Shanghai, China; 3 Center for Molecular Medicine, Children’s Hospital of Fudan University, National Children’s Medical Center, Shanghai, China; 4 Department of Otolaryngology–Head and Neck Surgery, Children’s Hospital of Fudan University, National Children’s Medical Center, Shanghai, China; 5 Key Laboratory of Birth Defects, Children’s Hospital of Fudan University, National Children’s Medical Center, Shanghai, China; 6 Key Laboratory of Neonatal Diseases, Ministry of Health, Children’s Hospital of Fudan University, National Children’s Medical Center, Shanghai, China

## Abstract

**Importance:**

Hearing loss is a global social burden. Early identification of hearing loss missed by newborn hearing screening tests in the neonatal intensive care unit is crucial.

**Objective:**

To assess the association between expanded genomic sequencing combined with hearing screening and detection of hearing loss as well as improvement in the neonatal intensive care unit.

**Design, Setting, and Participants:**

This cohort study was performed between August 8, 2016, and December 31, 2020, among 8078 newborns admitted to the neonatal intensive care unit of the Children’s Hospital of Fudan University in Shanghai, China. Follow-up for hearing status was performed via telephone interviews between September 1 and November 30, 2021.

**Exposures:**

A hearing screening test and the expanded genomic sequencing targeting 2742 genes were administered to each patient. Those who failed the hearing screening test or had positive genetic findings were referred for diagnostic audiometry at a median of 3 months of age.

**Main Outcomes and Measures:**

The primary outcome was hearing loss missed by hearing screening test. Secondary outcomes were genetic findings and benefits associated with the expanded genomic sequencing for clinical management of patients in the neonatal intensive care unit.

**Results:**

Of 8078 patients (4666 boys [57.8%]; median age, 6.3 days [IQR, 3.0-12.0 days]), 52 of 240 (21.7%) received a diagnosis of hearing loss. Expanded genomic sequencing combined with hearing screening was associated with a 15.6% increase (7 of 45 patients) in cases of diagnosed hearing loss that were missed by hearing screening. Of the 52 patients with hearing loss, genetic factors were identified for 39 patients (75.0%); *GJB2* and *SLC26A4* were the most common genes identified. Patients with genetic findings experienced a more severe degree of hearing loss than those without genetic findings (21 profound, 4 severe, 7 moderate, and 7 mild vs 2 severe, 4 moderate, and 7 mild; *P* = .005), with more bilateral hearing loss (39 of 39 [100%] vs 9 of 13 [69.2%]; *P* = .003). Clinical management strategies were changed for patients who underwent genomic sequencing combined with hearing screening.

**Conclusions and Relevance:**

This study suggests that expanded genomic sequencing combined with hearing screening may be effective at detecting hearing loss among patients in the neonatal intensive care unit.

## Key Points

**Question:**

Is expanded genomic sequencing combined with hearing screening associated with the detection of hearing loss and the improvement in the clinical management of patients in the neonatal intensive care unit (NICU)?

**Findings:**

In this cohort study that included 8078 patients in the NICU, expanded genomic sequencing was associated with a 15.6% increase in cases of diagnosed hearing loss that were missed by hearing screening and changed the clinical management strategies of these patients. Of 52 patients with a diagnosis of hearing loss, 39 (75%) had genetic findings and experienced a more severe degree of hearing loss.

**Meaning:**

This study suggests that expanded genomic sequencing combined with hearing screening may be effective for diagnosing hearing loss in the NICU setting.

## Teaser

This cohort study assesses the association between expanded genomic sequencing combined with hearing screening and the detection of hearing loss and improvement in the clinical management of patients in the neonatal intensive care unit.

## Introduction

Hearing loss, including sensorineural and conductive hearing loss, is a common complication among newborns worldwide.^1^ The incidence is estimated to be from 0.1% to 0.3% among healthy babies and increases to 2% to 4% among patients admitted to the neonatal intensive care unit (NICU).^2,3^ In 2015, hearing loss became the fourth major cause of disability; it ranked 11th in 2010, becoming a social burden globally.^4,5,6^ Early intervention can prevent subsequent damage to hearing, including impairment of cognitive function, language development, communication, and social-emotional development.^7,8,9^ Currently, the most cost-effective approach to prevent hearing loss is early screening and identification of the cause.^9,10^

Studies have indicated that, among newborns with hearing loss, those admitted to the NICU are thought to be at high risk, comprising 1.2% to 7.5% of cases of hearing loss.^9^ Currently, there are different protocols for hearing screening indicated for children admitted to the NICU.^7^ Previous studies have shown that approximately 25% of cases of hearing loss are missed by existing newborn hearing screening (NBHS) programs, wherein two-thirds receive a diagnosis of severe to profound hearing loss later.^11,12^ Previous studies have indicated that hearing loss among newborns in the NICU was associated not only with clinical risk factors, such as cytomegalovirus infection, craniofacial malformation, family history of hearing loss, duration of NICU stay, oxygen exposure, or low birth weight,^13,14,15,16,17,18,19,20^ but also with genetic disorders, which have been shown with the wider use of genetic testing to be associated with hearing loss.^21^ Microarray or limited genomic sequencing were used in screening for underlying hearing loss in the general neonatal population because of its low cost.^22,23,24,25^

Little is known regarding the use of genetic sequencing combined with NBHS programs for the screening of hearing loss in newborns who stayed in the NICU. We assessed the association between expanded genomic sequencing (EGS) combined with NBHS and the early detection of hearing loss among newborns admitted to the NICU in China for better management strategies of patients with this type of hearing loss.

## Methods

### Study Design and Data Collection

This cohort study retrospectively included 8078 newborns who were admitted to the Children’s Hospital of Fudan University in Shanghai, China, and underwent genetic testing in the China Neonatal Genomes Project^26^ between August 8, 2016, and December 31, 2020. The inclusion criteria were as follows: newborns admitted to NICU levels 3 and 4 (included but not limited to those requiring continuous monitoring [eg, respiration, heart rate, blood gas analysis, and cerebral function], ventilation, hypothermia therapy, gastroscopy, bronchoscopy, or extracorporeal membrane oxygenation) who underwent NBHS. The exclusion criteria were deceased newborns or newborns with insufficient information in the medical records. Patients who tested positive for hearing loss with the NBHS program or had positive genetic findings through EGS were referred for diagnostic audiometry and follow-up. Approval was obtained from the ethics committee of the Children’s Hospital of Fudan University. The parents of each patient provided written informed consent. This study followed the Strengthening the Reporting of Observational Studies in Epidemiology (STROBE) guideline.

For each patient, clinical information, NBHS findings, and genetic findings from the electronic medical record were independently obtained by 2 clinicians (Y.Z. and L.H.). Risk factors were recorded, which included the duration of NICU stay, oxygen exposure, low birth weight (<2500 g), maternal complications during pregnancy (including hypertensive disorders complicating pregnancy, gestational diabetes, or anemia during pregnancy), neonatal sepsis,^27^ perinatal asphyxia (including intrauterine hypoxia^28^ or birth asphyxia^29^), mechanical ventilation, craniofacial malformation, severe hyperbilirubinemia (total serum bilirubin level requires exchange transfusion), congenital cytomegalovirus infection (detection of cytomegalovirus in urine sample within 3 weeks after birth by polymerase chain reaction), neonatal bacterial meningitis,^30^ family history of hearing loss, and consanguineous parents.

### Newborn Hearing Screening

A hearing screening test was performed for each patient. Otoacoustic emission (Interacoustics OtoRead; Oticon Medical Inc) and automated auditory brainstem response (ALGO 3i screener; Natus Medical Inc) were tested successively by trained audiologists when the patients had stable conditions during admission. Infants who tested positive for hearing loss with the NBHS program were defined as those with positive NBHS results after testing for otoacoustic emission, automated auditory brainstem response, or both unilaterally or bilaterally.

### Genetic Sequencing and Interpretation

Expanded genomic sequencing was performed for every patient. Genomic DNA was extracted from whole-blood samples using a QIAamp DNA Blood Mini Kit (Qiagen). Fragments of DNA were enriched using the Agilent ClearSeq Inherited Disease panel kit (Agilent Technologies) that targeted 2742 genes. Sequencing was performed on a HiSeq 2000/2500 platform (Illumina Inc). The TruSeq Rapid PE Cluster and SBS Kits (Illumina Inc) were used for sequencing. Data analysis was conducted using an in-house pipeline.^31^ A virtual panel including nonsyndromic and syndromic hearing loss–related genes on the Hereditary Hearing Loss Homepage^32^ or in the Online Mendelian Inheritance in Man database^33^ were analyzed. Patients with pathogenic or likely pathogenic variants consistent with inheritance patterns received positive genetic findings based on the guidelines of the American College of Medical Genetics and Genomics.^34^ Sanger sequencing was performed to confirm the variant.

### Follow-up Strategy

Patients who tested positive for hearing loss with the NBHS program or had positive genetic findings were referred for diagnostic audiometry and audiologic counseling by audiologists. Hearing monitoring was arranged if necessary. Follow-up telephone interviews were performed between September 1 and November 30, 2021. Hearing status at the time of the telephone interview was recorded, including diagnosis of hearing loss, degree of hearing loss (mild, moderate, severe, or profound), symptoms (difficulty in hearing, communication, or speech), risk factors,^13,14,15,17,18,19,20^ and treatment (cochlear implant, hearing aids, or speech therapy).

### Diagnosis of Hearing Loss

Hearing loss diagnosed by trained audiologists was defined as a diagnostic audiology test result with a hearing threshold over 25 dB unilaterally or bilaterally and diagnosis of hearing loss by audiologists according to behavioral audiometry (patients ≥6 months of age). The degree of hearing loss according to the latest diagnostic audiometry result was classified as mild (hearing threshold, 26-30 dB), moderate (hearing threshold, 31-60 dB), severe (hearing threshold, 61-80 dB), or profound (hearing threshold, ≥81 dB).^35^

### Statistical Analysis

Continuous variables with a skewed distribution are reported as median values with interquartile ranges (IQRs). Categorical variables are expressed as frequencies and percentages. The Pearson χ^2^ test or the Fisher exact test was performed for comparative analysis. All *P* values were from 2-sided tests, and results were deemed statistically significant at *P* < .05. Statistical analysis was performed using Stata, version 15 (StataCorp LLC).

## Results

### Study Population

Of 8078 patients included in the study, the median age at admission was 6.3 days (IQR, 3.0-12.0 days), and 4666 (57.8%) were boys (Table 1; Figure). A total of 3322 patients (41.1%) were preterm, and 2947 (36.5%) had low birth weight.

**Table 1.** Characteristics of 8078 Patients

Characteristic	Patients, No. (%) (N = 8078)
Sex	
Male	4666 (57.8)
Female	3412 (42.2)
Gestational age	
<28 wk and 0 d	278 (3.4)
28 wk and 0 d to 31 wk and 6 d	1012 (12.5)
32 wk and 0 d to 36 wk and 6 d	2032 (25.2)
≥37 wk and 0 d	4756 (58.9)
Birth weight, g	
<1000	234 (2.9)
1000-1499	848 (10.5)
1500-2499	1865 (23.1)
2500-3999	4838 (59.9)
≥4000	293 (3.6)
Cesarean delivery	4117 (51.0)
Positive NBHS test result	238 (2.9)
Positive genetic findings^a^	90 (1.1)
Diagnosis of hearing loss	52 (0.6)

Abbreviation: NBHS, newborn hearing screening.

^a^ Patients identified as having hearing loss–related genes.

**Figure.**
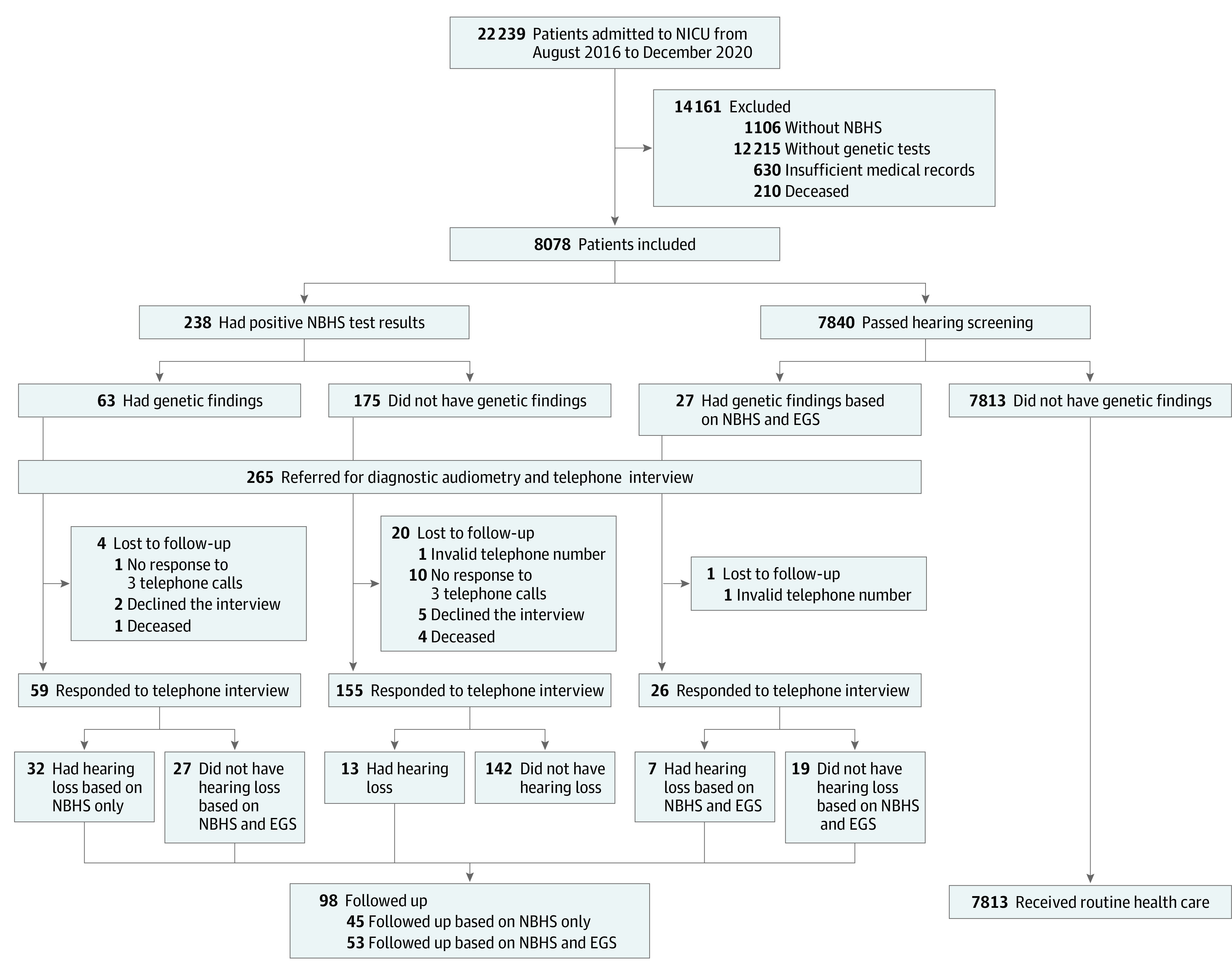
Study Profile and Benefits of Expanded Genomic Sequencing (EGS) Combined With Newborn Hearing Screening (NBHS) Program Associated With Referral, Diagnosis, and Clinical Management NICU indicates neonatal intensive care unit.

### Patients Referred Because of NBHS and EGS Results

Of 8078 patients, 238 (2.9%) tested positive for hearing loss with the NBHS program, while 7840 (97.1%) tested negative. The top 5 risk factors associated with hearing loss among the 238 patients who did not pass the NBHS test were hospitalization in the NICU for 5 days or more (210 [88.2%]), oxygen exposure (71 [29.8%]), maternal complications during pregnancy (69 [29.0%]), low birth weight (67 [28.2%]), and sepsis (62 [26.1%]) (eTable 1 in the Supplement).

In total, 90 of 8078 patients (1.1%) received a diagnosis of a genetic condition associated with 15 genes (eTable 2 in the Supplement). *GJB2* (OMIM 121011) was identified in 58 patients (64.4%) and *SLC26A4* (OMIM 605646) was identified in 17 patients (18.9%). Of these 90 patients, 70.0% (63 of 90) had positive NBHS test results. The remaining 30.0% patients (27 of 90) had negative NBHS test results and had positive genetic findings. Among those patients, *GJB2*:c.109G>A was the most prevalent variant (eTable 3 in the Supplement). In all, 265 patients (3.3%) were referred for diagnostic audiometry owing to postive NBHS test results or positive genetic findings.

### Patients With a Clinical Diagnosis of Hearing Loss

Of the 265 patients referred, 240 (90.6%) reported their hearing status after the telephone interview, while 25 (9.4%) were lost to follow-up (eTable 4 in the Supplement). In total, 52 of 240 patients (21.7%) developed hearing loss. The median age at diagnosis was 3 months (range, 1.5-48 months) (Table 2). Of 52 patients with a diagnosis of hearing loss, 39 (75.0%) had genetic findings associated with 8 genes. *GJB2* and *SLC26A4* were identified in 33 of these 39 patients and accounted for 84.6% of cases. *GJB2*:c.109G>A had the highest allele frequency (5.8%) in the cohort. The allele frequency of *GJB2*:c.109G>A was higher among patients with a diagnosis of hearing loss than among those without hearing loss (12 of 104 [11.5%] vs 929 of 16 052 [5.8%]; *P* = .01). Among these patients with positive genetic findings, the degree of hearing loss was more severe (21 profound, 4 severe, 7 moderate, and 7 mild) compared with patients without genetic findings (2 severe, 4 moderate, and 7 mild; *P* = .005). More patients with genetic findings than without genetic findings received a diagnosis of bilateral hearing loss (39 of 39 [100%] vs 9 of 13 [69.2%]; *P* = .003). However, the median age at onset (0 months [range, 0-0 months] vs 0 months [range, 0-42 months]; *P* = .80) and the median age at diagnosis (3 months [range, 3-30 months] vs 3 months [range, 1.5-48 months]; *P* = .80) were not significantly different between patients with positive genetic findings and those without genetic findings.

**Table 2.** Genetic and Hearing Screening Results From 52 Patients With a Diagnosis of Hearing Loss

Characteristic	Patients, No./total No. (%)			
	Positive NBHS test result and genetic findings (n = 63)	Negative NBHS test result and genetic findings (n = 27)	Positive NBHS test result without genetic findings (n = 175)	Total (N = 265)
Response to interview, No. (%)	59 (93.7)	26 (96.3)	155 (88.6)	240 (90.6)
Age at interview, median (range), mo	33 (9-64)	33.5 (12-67)	30 (9-61)	30.5 (9-67)
Age at diagnosis, median (range), mo	3 (1.5-24)	24 (6-48)	3 (3-30)	3 (1.5-48)
Hearing loss	32/59 (54.2)	7/26 (26.9)	13/155 (8.4)	52/240 (21.7)
Degree^a^				
Mild	7/32 (21.9)	0	7/13 (53.8)	14/52 (26.9)
Moderate	7/32 (21.9)	0	4/13 (30.8)	11/52 (21.2)
Severe	2/32 (6.2)	2/7 (28.6)	2/13 (15.4)	6/52 (11.5)
Profound	16/32 (50.0)	5/7 (71.4)	0	21/52 (40.4)
Laterality				
Unilateral	0	0	4/13 (30.8)	4/52 (7.7)
Bilateral	32/32 (100)	7/7 (100)	9/13 (69.2)	48/52 (92.3)
Treatment^b^				
Cochlear implant	11/32 (34.4)	2/7 (28.6)	1/13 (7.7)	14/52 (26.9)
Hearing aids	11/32 (34.4)	5/7 (71.4)	2/13 (15.4)	18/52 (34.6)
Speech therapy	7/32 (21.9)	2/7 (28.5)	1/13 (7.7)	10/52 (19.2)
None	13/32 (40.6)	0	10/13 (76.9)	23/52 (44.2)

Abbreviation: NBHS, newborn hearing screening.

^a^ Indicating patients with hearing loss in this subgroup. For patients with different degrees of hearing in each ear, only the ear with the more severe hearing loss was counted.

^b^ Indicating patients with treatment of hearing loss in this subgroup. The different types of treatment in 1 patient were counted separately.

Of 63 patients who had positive NBHS test results and positive genetic findings, 59 completed the follow-up. Of these 59 patients, 32 (54.2%) were confirmed to have mild to profound hearing loss bilaterally with early diagnosis (eTable 5 in the Supplement).

Of 27 patients who had negative NBHS test results but positive genetic findings, 26 completed the follow-up, 7 of whom (26.9%) received a diagnosis of severe to profound hearing loss (Table 3). Of these 7 patients, 6 (85.7%) with variants in *SLC26A4* received a diagnosis of enlargement of vestibular aqueduct at a median age of 24 months (range, 9-48 months). One patient (14.3%) with *GJB2*:c.235del received a diagnosis at 6 months of age.

**Table 3.** Results of Follow-up in Patients Who Passed Hearing Screening and Had Positive Genetic Findings

Gene and variant(s)	Zygosity	Age at diagnosis, median (range), mo	Hearing loss, No.^a^			Treatment, No.			No hearing loss, No.	Total, No.
			M/M	S/P	Total	Cochlear implant	Hearing aids or speech therapy	None		
*GJB2*										
c.109G>A	Hom	NA	NA	NA	NA	NA	NA	NA	10	10
c.109G>A /c.235del	Het/het	NA	NA	NA	NA	NA	NA	NA	3	3
c.235del	Hom	6	0	1	1	1	0	0	1	2
*SLC26A4*										
c.589G>A/c.1174A>T	Het/het	42	0	1	1	0	1	0	0	1
c.919-2A>G	Hom	10.5 (9-12)	0	2	2	1	1	0	0	2
c.919-2A>G/c.1975G>C	Het/het	48	0	1^b^	1	0	1	0	0	1
c.919-2A>G/c.2168A>G	Het/het	24	0	1	1	0	1	0	0	1
c.1226G>A	Hom	39	0	1	1	0	1	0	0	1
c.1229C>T/c.2168A>G	Het/het	NA	NA	NA	NA	NA	NA	NA	1	1
c.2168A>G	Hom	NA	NA	NA	NA	NA	NA	NA	1	1
*COL11A1*										
c.3816 + 2dup	Het	NA	NA	NA	NA	NA	NA	NA	1	1
*KCNQ4*										
c.2039C>T	Het	NA	NA	NA	NA	NA	NA	NA	1	1
*MAF*										
c.161C>T	Het	NA	NA	NA	NA	NA	NA	NA	1	1
Total	NA	24 (6-48)	0	7	7	2	5	0	19	26

Abbreviations: Het, heterozygosity; Hom, homozygosity; M/M, mild or moderate degree of hearing loss; NA, not applicable; S/P, severe or profound degree of hearing loss.

^a^ For patients with different degrees of hearing in each ear, only the ear with the more severe hearing loss was counted.

^b^ The degree of hearing loss in patient 152 progressively increased from 85 dB in the left ear and 60 dB in the right ear to 85 dB in the left ear and 95 dB in the right ear.

Of 175 patients who had positive NBHS test results but did not have genetic findings, 155 completed the follow-up. A total of 13 patients (8.4%) received a diagnosis of mild to severe hearing loss, including 4 patients with unilateral hearing loss (eTable 6 in the Supplement). Most patients in this subgroup had multiple risk factors (median, 3 risk factors; range, 1-8 risk factors). Compared with those who had bilateral hearing loss, patients with unilateral hearing loss showed no significant differences in the degree of hearing loss (1 moderate and 3 mild vs 2 severe, 3 moderate, and 4 mild; *P* = .49) or in the number of risk factors (median, 2.5 [range, 1-4] vs median, 3 [range, 1-8]; *P* = .48).

### Patients Without Clinical Diagnosis of Hearing Loss by Diagnostic Audiologists

At the last follow-up, 188 of 240 patients (78.3%) did not receive a diagnosis of hearing loss. Of these 188 patients, 46 had positive genetic findings, including 27 patients who tested positive after the NBHS and 19 patients who tested negative. The 46 patients who had positive genetic findings were identified with genetic findings of homozygous or heterozygous *GJB2*:c.109G>A in 34 patients (73.9%), *GJB2*:c.235del in 1 patient (2.2%), *SLC26A4* variants in 3 patients (6.5%), *COL11A1* (OMIM 120280) in 2 patients (4.3%), and variants in *COL2A1* (OMIM 120140), *CREBBP* (OMIM 600140), *FLNA* (OMIM 300017), *KCNQ4* (OMIM 603537), *MAF* (OMIM 177075), and *PTPN11* (OMIM 176876)*,* each of which was seen in 1 patient (Table 3; eTable 5 in the Supplement). These patients at risk for hearing loss were followed up.

### EGS Combined With NBHS

Expanded genomic sequencing combined with NBHS had a beneficial association with the referral, diagnosis, and clinical management of the newborns in this study. Of 8078 patients, 238 were referred for diagnostic audiometry if only NBHS was performed. For those who underwent both EGS and NBHS, an additional 27 patients at risk for hearing loss were identified; they had positive genetic findings but had negative NBHS test results. Of 52 patients with a diagnosis of hearing loss, 45 were diagnosed through the NBHS program only. However, EGS combined with NBHS identified an additional 15.6% of patients (7 of 45) with hearing loss who were missed by the NBHS program. The increased diagnosis rate was 0.09% (7 of 8078) among the whole cohort. If only NBHS was performed, 45 patients with a diagnosis of hearing loss were followed up. A total of 98 patients (52 patients with hearing loss and 46 patients who had genetic findings but without hearing loss) who underwent EGS and NBHS were followed up (Figure). Of the 52 patients with a diagnosis of hearing loss, 39 (75.0%) had genetic factors that were identified.

## Discussion

In this study, we assessed EGS combined with NBHS for 8078 patients in the NICU who were at high risk for hearing loss. We found that EGS combined with NBHS was associated with an increase in the referral rate and in the number of cases of hearing loss diagnosed that were missed by NBHS, as well as changes in the clinical management of patients with the possibility of hearing loss. Of the patients with a diagnosis of hearing loss, 75.0% (39 of 52) clarified the genetic findings presented with a more severe degree of hearing loss bilaterally. Such patients need early treatment to improve their prognosis. Expanded genomic sequencing combined with NBHS may be useful in identifying patients with underlying hearing loss who need proper clinical management and/or changes in clinical management.

Newborn hearing screening programs have been shown to have significant benefits worldwide.^36,37^ Some studies have shown that the referral rate by NBHS only in the general neonatal population ranges from 0.4% to 3.2%,^23,25,38,39^ while it is 3.9% among the NICU population.^19^ Both NBHS and genetic testing were associated with a 0.2% to 0.9% increase in the referral rate among the general population.^18,23,38^ Our study reported a referral rate of 2.9% with NBHS only; however, the combination of EGS and NBHS was associated with an increase of 0.4%. Therefore, EGS combined with NBHS was associated with the identification of these referred patients at possible risk for hearing loss.

Genetic testing identified cases of underlying hearing loss that were missed by the NBHS. Some studies have demonstrated that genetic tests were associated with an increase in the number of additional cases of hearing loss that were missed by the NBHS by 0.005% to 0.05%.^23,25^ In our study, EGS combined with NBHS was associated with a 15.6% increase in the hearing loss diagnosis rate, which was also associated with an additional 0.09% increase in the hearing loss diagnosis rate among the whole cohort. Our diagnosis rate was higher than in previous studies. In our study, we used EGS with a broader detection range compared with commonly used microarray methods in China, including 9 to 20 variants in several hearing genes.^22,23^ Expanded genomic sequencing detected the entire coding region, including variants uncovered by microarray. In addition, patients in NICU levels 3 and 4 had a higher incidence of genetic diseases compared with the general neonatal population. Therefore, EGS combined with NBHS was associated with an increased diagnosis rate among this population.

Exome sequencing presented an advantage in the early diagnosis of hearing loss^12,18,40,41^ and of other genetic disorders.^42,43,44,45,46^ However, the high cost and long turnaround time limit the applicability of newborn genetic screening. We performed EGS on an NICU cohort, underscoring its effectiveness. Our turnaround time was 20 days, and the cost of EGS was $250.^47^ With the shortened duration and the decreasing cost of sequencing, EGS combined with NBHS presents a promising possibility for genetic testing in the NICU setting.

Expanded genomic sequencing plus NBHS is also associated with benefits for clinical management. It was reported that genetic factors account for 50% to 60% of congenital hearing loss cases.^21,48,49^ Of 52 patients with a diagnosis of hearing loss in our study, we noted that 75.0% had confirmed genetic findings and presented with a more severe degree of bilateral hearing loss. Such patients need early treatment for a better prognosis. In addition, all patients with unilateral hearing loss were identified among the group that had positive NBHS test results but did not have genetic findings. More patients are needed to delineate the clinical characteristics of unilateral hearing loss.

Of 188 patients without hearing loss up to the last follow-up, 46 had genetic findings. Among these 46 patients, homozygous or heterozygous *GJB2*:c.109G>A was mostly prevalent, together with other variants associated with progressive, late-onset hearing loss (*COL11A1*, *GJB2*:c.235del, *KCNQ4*, *MAF*, and *SLC26A4*) or syndromes partially characterized by hearing loss (*COL11A1*, *COL2A1*, *CREBBP*, *FLNA*, and *PTPN11*).

*GJB2*:c.109G>A is a hotspot variant in East Asia, with an allele frequency of 8.4%. Patients with c.109G>A variants may develop progressive hearing loss owing to variable expressivity and incomplete penetrance.^38,49,50,51,52^ The ages at onset of homozygous or heterozygous c.109G>A range from infancy to 30 years. Individuals with homozygous c.109G>A lose hearing at a rate of 1 dB per year, with penetrance of 17% by young adulthood in the Chinese population.^38,51,53^ Of 31 patients with homozygous c.109G>A in our cohort, 6 (19.4%) developed hearing loss up to our last follow-up. This finding is consistent with previous data.^51^ Currently, c.109G>A is not detected in neonatal genetic screening for hearing in China. Underlying hearing loss may be missed among patients with this variant. We think that c.109G>A should be included in genetic screening for hearing among newborns. Patients with c.109G>A should be followed up for the progression of hearing loss among those with a diagnosis of hearing loss and for the occurrence among those without hearing loss.

*GJB2*:c.235del, which is associated with moderate to profound congenital hearing loss, is another frequent variant in the Chinese population. A previous study reported patients with homozygous or heterozygous *GJB2*:c.235del whose hearing loss was missed by otoacoustic emission or automated auditory brainstem response testing, which could delay a diagnosis and may affect the prognosis.^54^ Therefore, EGS is helpful for the early diagnosis and management of late-onset hearing loss for this type of patient. Patients with variants in *SLC26A4*, which may lead to sudden hearing loss after head trauma or barotrauma, were strongly advised to avoid situations that may result in sudden changes in intracranial pressure, to prevent or delay the occurrence of hearing loss.^55^

Expanded genomic sequencing combined with NBHS is useful for diagnosing hearing loss, managing patients, monitoring progression, genetic counseling, and educating patients on the prevention of precipitating factors. It also highlights the importance of follow-up for preventing or delaying the occurrence of hearing loss and of early diagnosis for prompt treatment of late-onset hearing loss if it occurs.

### Limitations

This study had several limitations. Our single-center cohort was generated from the China Neonatal Genomes Project, which may have led to selection bias. Moreover, patients with a limited follow-up period needed a longer time for monitoring the occurrence of hearing loss. We also did not analyze the cost-effectiveness of the procedure. In the future, multicenter studies will need to be performed for the development of an effective genetic testing strategy for the NICU population.

## Conclusions

The findings of this cohort study suggest that EGS combined with NBHS was associated with an increase in the referral rate and in the number of cases of underlying hearing loss missed by NBHS and that the genetic factors associated with hearing loss need to be identified for the proper clinical management of hearing loss in patients. Genetic factors were associated with hearing loss in the patients in the NICU. We recommend EGS combined with NBHS for hearing loss diagnosis in the NICU setting.

## Notes

Supplement. **eTable 1.** Risk Factors of Hearing Loss in 238 Patients With Failed Hearing Screening **eTable 2.** Genes With Pathogenic/Likely Pathogenic Variants Identified in 8078 Patients **eTable 3.** Results of Genetic Tests and Relationship With Hearing Screening in 8078 Patients **eTable 4.** Characteristics of 25 Patients Lost to Follow-up Compared With 240 Patients Who Completed Follow-up **eTable 5.** Results of Follow-ups in Patients Who Failed Hearing Screening and Had Genetic Findings **eTable 6.** Hearing Loss Diagnosed Without Genetic Findings
