# Supplementary material for: Association Between Expanded Genomic Sequencing Combined With Hearing Screening and Detection of Hearing Loss Among Newborns in a Neonatal Intensive Care Unit
Source: JAMA Netw Open. 2022 Jul 11;5(7):e2220986. doi: 10.1001/jamanetworkopen.2022.20986 (PMC9274323; doi:10.1001/jamanetworkopen.2022.20986)
Supplement: Supplement. — eTable 1. Risk Factors of Hearing Loss in 238 Patients With Failed Hearing Screening eTable 2. Genes With Pathogenic/Likely Pathogenic Variants Identified in 8078 Patients eTable 3. Results of Genetic Tests and Relationship With Hearing Screening in 8078 Patients eTable 4. Characteristics of 25 Patients Lost to Follow-up Compared With 240 Patients Who Completed Follow-up eTable 5. Results of Follow-ups in Patients Who Failed Hearing Screening and Had Genetic Findings eTable 6. Hearing Loss Diagnosed Without Genetic Findings [file jamanetwopen-e2220986-s001.pdf]

Supplementary Online Content

Zhu Y, Hu L, Yang L, et al. Association between expanded genomic sequencing combined with hearing screening and detection of hearing loss among newborns in a neonatal intensive care unit. *JAMA Netw Open.* 2022;5(7):e2220986. doi:10.1001/jamanetworkopen.2022.20986

eTable 1. Risk Factors of Hearing Loss in 238 Patients With Failed Hearing Screening
eTable 2. Genes With Pathogenic/Likely Pathogenic Variants Identified in 8078 Patients
eTable 3. Results of Genetic Tests and Relationship With Hearing Screening in 8078 Patients
eTable 4. Characteristics of 25 Patients Lost to Follow-up Compared With 240 Patients Who Completed Follow-up
eTable 5. Results of Follow-ups in Patients Who Failed Hearing Screening and Had Genetic Findings
eTable 6. Hearing Loss Diagnosed Without Genetic Findings

This supplementary material has been provided by the authors to give readers additional information about their work.

© 2022 Zhu Y et al. *JAMA Network Open.*

eTable 1. Risk Factors of Hearing Loss in 238 Patients With Failed Hearing Screening

Risk factors	No. (%)[a]
NICU stay ≥ 5 days	210 (88.2)
Oxygen exposure	71 (29.8)
Maternal complication during pregnancy	69 (29.0)
Low birth weight	67 (28.2)
Sepsis	62 (26.1)
Perinatal asphyxia	50 (21.0)
Mechanical ventilation	45 (18.9)
Craniofacial malformation	29 (12.2)
Severe hyperbilirubinemia	20 (8.4)
Cytomegalovirus infection	14 (6.4)[b]
Bacterial meningitis	10 (4.2)
Family history of hearing loss	4 (1.7)
Consanguineous parents	1 (0.4)

[a] Multiple risk factors in one patient were counted separately. [b] A total of 220 of 238 patients conducted tests for cytomegalovirus.

© 2022 Zhu Y et al. *JAMA Network Open.*

eTable 2. Genes With Pathogenic/Likely Pathogenic Variants Identified in 8078 Patients

Gene	Disease [MIM number]	Inheritance	No. (%)[a]
GJB2	Deafness, autosomal recessive 1A [220290]	AR/DD	58 (64.4)
SLC26A4	Deafness, autosomal recessive 4, with enlarged vestibular aqueduct [600791], Pendred syndrome [274600]	AR	17 (18.9)
COL11A1	Deafness, autosomal dominant 37 [618533], Marshall syndrome [154780], Stickler syndrome, type II [604841]	AD	2 (2.2)
COL2A1	Kniest dysplasia [156550], SED congenita [183900], Spondyloperipheral dysplasia [271700], Stickler syndrome, type I [108300]	AD	2 (2.2)
CHD7	CHARGE syndrome [214800], Hypogonadotropic hypogonadism 5 with or without anosmia [612370]	AD	1 (1.1)
CREBBP	Menke-Hennekam syndrome 1 [618332], Rubinstein-Taybi syndrome 1 [180849]	AD	1 (1.1)
FLNA	Frontometaphyseal dysplasia 1[305620], Otopalatodigital syndrome, type I [311300], Otopalatodigital syndrome, type II [304120]	XLD/XLR	1 (1.1)
KCNQ4	Deafness, autosomal dominant 2A [600101]	AD	1 (1.1)
MAF	Ayme-Gripp syndrome [601088]	AD	1 (1.1)
MYH9	Deafness, autosomal dominant 17 [603622], Macrothrombocytopenia and granulocyte inclusions with or without nephritis or sensorineural hearing loss [155100]	AD	1 (1.1)
OTOGL	Deafness, autosomal recessive 84B [614944]	AR	1 (1.1)
PEX1	Heimler syndrome 1 [234580], Peroxisome biogenesis disorder 1A (Zellweger) [214100], Peroxisome biogenesis disorder 1B (NALD/IRD) [601539]	AR	1 (1.1)
PTPN11	LEOPARD syndrome 1 [151100], Noonan syndrome 1 [163950]	AD	1 (1.1)
RUNX2	Cleidocranial dysplasia [119600]	AD	1 (1.1)
TRRAP	Developmental delay with or without dysmorphic facies and autism [618454]	AD	1 (1.1)
**Total**	NA	NA	90 (100)

Abbreviations: AD, autosomal dominant inheritance; AR, autosomal recessive inheritance; DD, digenic dominant

© 2022 Zhu Y et al. *JAMA Network Open.*

inheritance; NA, not applicable; XLD, X-linked dominant inheritance; XLR, X-linked recessive inheritance. [a] Percentage was the proportion of the gene in all genes.

© 2022 Zhu Y et al. *JAMA Network Open.*

eTable 3. Results of Genetic Tests and Relationship With Hearing Screening in 8078 Patients

Gene	Variant(s)	Zygosity	Hearing screening, No. (%)		Total
			**Positive**	**Negative**	
GJB2	c.109G>A	Hom	23 (68)	11 (32)	34
	c.109G>A/c.176_191del	Het/Het	1 (100)	0 (0)	1
	c.109G>A /c.235del	Het/Het	5 (63)	3 (38)	8
	c.109G>A/c.299_300del	Het/Het	1 (100)	0 (0)	1
	c.139G>T/c.235del	Het/Het	1 (100)	0 (0)	1
	c.176_191del/c.235del	Het/Het	1 (100)	0 (0)	1
	c.235del	Hom	8 (80)	2 (20)	10
	c.235del/c.299_300del	Het/Het	1 (100)	0 (0)	1
	c.299_300del	Hom	1 (100)	0 (0)	1
SLC26A4	c.589G>A/c.1174A>T	Het/Het	1 (50)	1 (50)	2
	c.600+2T>A/c.919-2A>G	Het/Het	1 (100)	0 (0)	1
	c.919-2A>G	Hom	2 (50)	2 (50)	4
	c.919-2A>G/c.1343C>T	Het/Het	1 (100)	0 (0)	1
	c.919-2A>G/c.1707+5G>A	Het/Het	1 (100)	0 (0)	1
	c.919-2A>G/c.1975G>C	Het/Het	0 (0)	1 (100)	1
	c.919-2A>G/c.2168A>G	Het/Het	1 (50)	1 (50)	2
	c.1174A>T/c.1229C>T	Het/Het	1 (100)	0 (0)	1
	c.1226G>A	Hom	0 (0)	1 (100)	1
	c.1229C>T/c.1975G>C	Het/Het	1 (100)	0 (0)	1
	c.1229C>T/c.2168A>G	Het/Het	0 (0)	1 (100)	1
	c.2168A>G	Hom	0 (0)	1 (100)	1
COL2A1	c.2050-1G>C	Het	1 (100)	0 (0)	1
	c.2818C>T	Het	1 (100)	0 (0)	1
COL11A1	c.1798C>T	Het	1 (100)	0 (0)	1
	c.3816+2dup	Het	0 (0)	1 (100)	1
CHD7	c.6165_6166del	Het	1 (100)	0 (0)	1
CREBBP	c.4660A>T	Het	1 (100)	0 (0)	1
FLNA	c.620C>T	Het	1 (100)	0 (0)	1
KCNQ4	c.2039C>T	Het	0 (0)	1 (100)	1
MAF	c.161C>T	Het	0 (0)	1 (100)	1
MYH9	c.534dup	Het	1 (100)	0 (0)	1
OTOGL	c.2833C>T/c.2911del	Het/Het	1 (100)	0 (0)	1
PEX1	c.2050C>T/c.3043G>T	Het/Het	1 (100)	0 (0)	1
PTPN11	c.1510A>G	Het	1 (100)	0 (0)	1
RUNX2	c.860-1G>C	Het	1 (100)	0 (0)	1
TRRAP	c.3316G>A	Het	1 (100)	0 (0)	1
**Total**	/	/	63 (70)	27 (30)	90

Abbreviations: Het, heterozygous variants; Hom, homozygous variants.

© 2022 Zhu Y et al. *JAMA Network Open.*

eTable 4. Characteristics of 25 Patients Lost to Follow-up Compared With 240 Patients Who Completed Follow-up

Characteristics	Lost to follow-up, No. (%) (n=25)	Completed follow-up, No. (%) (n=240)	*P* value
**Sex**			
Male	14 (56.0)	153 (63.8)	.45
Female	11 (44.0)	87 (36.2)	
**Gestational age**			.26
< 28 weeks	1 (4.0)	7 (2.9)	
28-31 weeks	3 (12.0)	18 (7.5)	
32-36 weeks	8 (32.0)	44 (18.3)	
≥ 37 weeks	13 (52.0)	171 (71.3)	
**Birth weight**			.006
< 1000 g	1 (4.0)	6 (2.5)	
1000-1499 g	2 (8.0)	16 (6.7)	
1500-2499 g	11 (44.0)	36 (15.0)	
2500-3999 g	11 (44.0)	171 (71.2)	
≥ 4000 g	0 (0.0)	11 (4.6)	
**Cesarean**	9 (36.0)	104 (43.3)	.48
**Failed NBHS**	24 (96.0)	214 (89.2)	.49
**Positive genetic findings**[a]	5 (20.0)	85 (35.4)	.12

Abbreviations: NBHS, Newborn hearing screening programs. [a] Positive genetic findings were patients identified with hearing loss related genes.

© 2022 Zhu Y et al. *JAMA Network Open.*

eTable 5. Results of Follow-ups in Patients Who Failed Hearing Screening and Had Genetic Findings

Gene	Variant(s)	Zygosity	AI, *M* (range), (months)	AD, *M* (range), (months)	HL, No.[a]			Treatment, No.			Non-HL, No.	Total, No.
					M/M	S/P	Total	CI	HA/ST	None		
GJB2	c.109G>A	Hom	41 (9–64)	3 (3–6)	5	1	6	0	2	4	15	21
	c.109G>A/ c.176_191del	Het/ Het	20	NA	NA	NA	NA	NA	NA	NA	1	1
	c.109G>A/c.235del	Het/ Het	21.5 (10–26)	NA	NA	NA	NA	NA	NA	NA	4	4
	c.109G>A/ c.299_300del	Het/ Het	25	NA	NA	NA	NA	NA	NA	NA	1	1
	c.139G>T/c.235del	Het/ Het	22	4	1	0	1	0	0	1	0	1
	c.176_191del/ c.235del	Het/ Het	44	3	0	1	1	0	1	0	0	1
	c.235del	Hom	26.5 (15–62)	3 (1.5–4)	2	6	8	4	3	2	0	8
	c.235del/ c.299_300del	Het/Het	25	3	0	1	1	1	1	0	0	1
	c.299_300del	Hom	53	24	1	0	1	0	0	1	0	1
SLC26A4	c.589G>A/c.1174A>T	Het/Het	34	4.5	0	1	1	1	1	0	0	1
	c.600+2T>A/c.919-2A>G	Het/Het	64	18	0	1	1	0	1	0	0	1
	c.919-2A>G	Hom	49.5 (39–60)	8 (3–13)	0	2	2	2	1	0	0	2
	c.919-2A>G/c.1343C>T	Het/Het	27	3	1	0	1	0	1	0	0	1
	c.919-2A>G/c.1707+5G>A	Het/Het	57	3	0	1	1	1	1	0	0	1
	c.919-2A>G/c.2168A>G	Het/Het	28	3	0	1	1	1	1	0	0	1
	c.1174A>T/c.1229C>T	Het/Het	41	4	0	1	1	0	1	0	0	1
	c.1229C>T/c.1975G>C	Het/Het	47	NA	NA	NA	NA	NA	NA	NA	1	1
COL2A1	c.2050-1G>C	Het	47	NA	NA	NA	NA	NA	NA	NA	1	1
	c.2818C>T	Het	28	1.5	1	0	1	0	0	1	0	1
COL11A1	c.1798C>T	Het	29	NA	NA	NA	NA	NA	NA	NA	1	1
CREBBP	c.4660A>T	Het	55	NA	NA	NA	NA	NA	NA	NA	1	1
FLNA	c.620C>T	Het	29	NA	NA	NA	NA	NA	NA	NA	1	1

© 2022 Zhu Y et al. *JAMA Network Open.*

Gene	Variant(s)	Zygosity	AI, *M* (range), (months)	AD, *M* (range), (months)	HL, No.			Treatment, No.			Non-HL, No.	Total, No.
					M/M	S/P	Total	CI	HA/ST	None		
MYH9	c.534dup	Het	45	2.5	0	1	1	1	1	0	0	1
OTOGL	c.2833C>T/c.2911del	Het/Het	23	5	1	0	1	0	0	1	0	1
PEX1	c.2050C>T/c.3043G>T	Het/Het	46	5	0	1	1	0	0	1	0	1
PTPN11	c.1510A>G	Het	44	NA	NA	NA	NA	NA	NA	NA	1	1
RUNX2	c.860-1G>C	Het	23	3	1	0	1	0	0	1	0	1
TRRAP	c.3316G>A	Het	13	3	1	0	1	0	0	1	0	1
**Total**	NA	NA	33 (9–64)	3 (1.5–24)	14	18	32	11	15	13	27	59

Abbreviations: AD, age at diagnosis; AI, age at interview; CI, Cochlear implant; HA, Hearing aids; Het, heterozygosity; HL, hearing loss; Hom, homozygosity; M/M, mild or moderate degree of hearing loss; *M*, median; NA, not applicable; S/P, severe or profound degree of hearing loss; ST, Speech therapy; . [a] The different degree of hearing in both ears in one patient was counted according to the more severe one.

© 2022 Zhu Y et al. *JAMA Network Open.*

eTable 6. Hearing Loss Diagnosed Without Genetic Findings

No.	Sex	GA (weeks)	AI (months)	AD (months)	Main clinical diagnosis	Risk factors of hearing loss	Degree	Laterality	Treatment
P-19	M	40^{+0}	56	30	Cholestasis, cleft palate	NICU stay ≥ 5 d, perinatal asphyxia	Moderate	Bilateral	Hearing aids/ speech therapy
P-95	M	39^{+1}	38	3	Hyperbilirubinemia, congenital heart disease	Severe hyperbilirubinemia	Moderate/ severe	Bilateral	Hearing aids
P-118	F	37^{+5}	33	6	Congenital malformation (fish lip), intracranial hemorrhage, congenital heart disease	NICU stay ≥ 5 d, maternal complication during pregnancy, low birth weight, craniofacial malformation	Mild	Bilateral	None
P-233	M	35^{+5}	11	3	Hyperbilirubinemia, preterm, congenital heart disease	NICU stay ≥ 5 d	Mild	Unilateral	None
P-28	M	39^{+5}	53	3	Sepsis, lebanese fossa fistula	NICU stay ≥ 5 d, oxygen exposure, sepsis, mechanical ventilation	Mild	Bilateral	None
P-259	M	41^{+0}	10	3	Neonatal hypoglycemia, sepsis	NICU stay ≥ 5 d, sepsis, maternal complication during pregnancy	Mild	Unilateral	None
P-194	M	38^{+4}	27	3	Absence of eyeball (left eye), sepsis, craniofacial malformation	NICU stay ≥ 5 d, sepsis, craniofacial malformation, cytomegalovirus infections	Mild	Unilateral	None

© 2022 Zhu Y et al. *JAMA Network Open.*

No.	Sex	GA (weeks)	AI (months)	AD (months)	Main clinical diagnosis	Risk factors of hearing loss	Degree	Laterality	Treatment
P-243	F	40^{+0}	9	3	Seizure, congenital heart disease	NICU stay ≥ 5 d, oxygen exposure	Moderate	Bilateral	None
P-146	M	21^{+2}	31	3	Preterm, neonatal encephalopathy	NICU stay ≥ 5 d, oxygen exposure, maternal complication during pregnancy, low birth weight, sepsis, perinatal asphyxia, mechanical ventilation, craniofacial malformation, cytomegalovirus infections	Mild	Bilateral	None
P-162	F	38^{+3}	17	3	Small for gestational age, congenital dysplasia joint, subdural hemorrhage	NICU stay ≥ 5 d, maternal complication during pregnancy, low birth weight, craniofacial malformation	Moderate	Unilateral	None
P-169	F	40^{+6}	7	7	Pierre Robin Syndrome	NICU stay ≥ 5 d, oxygen exposure, maternal complication during pregnancy, low birth weight, perinatal asphyxia, mechanical ventilation, craniofacial malformation	Mild/ moderate	Bilateral	Non
P-197	M	39^{+0}	12	8	Hyperbilirubinemia, subdural hemorrhage	Maternal complication during pregnancy, craniofacial malformation	Moderate/ severe	Bilateral	None[a]

© 2022 Zhu Y et al. *JAMA Network Open.*

No.	Sex	GA (weeks)	AI (months)	AD (months)	Main clinical diagnosis	Risk factors of hearing loss	Degree	Laterality	Treatment
P-235	M	38^{+1}	12	3	Hyperbilirubinemia, intraventricular hemorrhage	NICU stay ≥ 5 d, maternal complication during pregnancy, craniofacial malformation	Mild	Bilateral	None

Abbreviations: AD, age at diagnosis; AI, age at interview; F, female; GA, gestational age; M, male;. [a] P-197 was suggested for unilateral cochlear implant after auricle reconstruction.

© 2022 Zhu Y et al. *JAMA Network Open.*
